# Aspiration After Sealing the Entrance by Stenting Is a Promising Method to Treat Subintimal Hematoma

**DOI:** 10.1016/j.jaccas.2021.01.004

**Published:** 2021-03-17

**Authors:** Akinori Sumiyoshi, Astunori Okamura, Mutsumi Iwamoto, Hiroyuki Nagai, Tomohiro Yamasaki, Takamasa Tanaka, Kohta Tanaka, Katusomi Iwakura, Kenshi Fujii

**Affiliations:** Cardiovascular Vascular Center, Sakurabashi Watanabe Hospital, Osaka, Japan

**Keywords:** acute coronary syndrome, complication, dissection, CAG, coronary angiography, IVUS, intravascular ultrasound, PCI, percutaneous coronary intervention, RCA, right coronary artery, STRAW, subintimal transcatheter withdrawa, TIMI, Thrombolysis In Myocardial Infarction

## Abstract

Creation of a distal re-entry site is widely performed to treat subintimal hematoma. However, this method has a risk of further vessel damage. The present aspiration technique after sealing the entry site by stenting is more promising because the hematoma can be reduced without additional vessel damage. (**Level of Difficulty: Advanced.**)

## Graphical abstract

**Figure:**
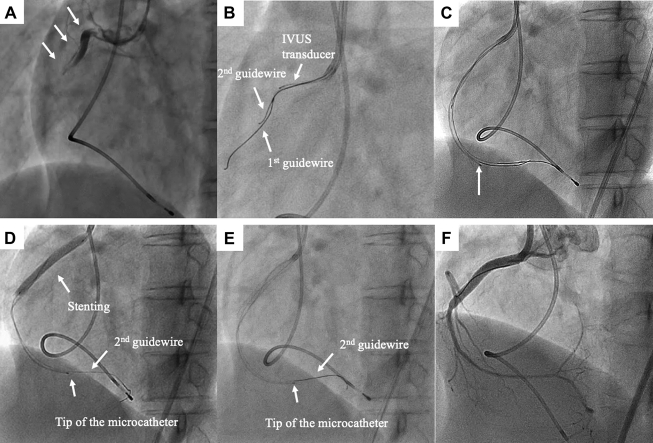

## Introduction

Subintimal hematoma of the coronary artery is caused by iatrogenic intimal damage, such as intimal damage during coronary angiography (CAG) or percutaneous coronary intervention (PCI). Subintimal hematoma is treated by sealing of the entry site by coronary stenting and then reducing the residual hematoma by creating a re-entry by means of guidewire fenestration or scoring balloon dilation at a site distal to the hematoma (1). However, the re-entry cannot always be created, and this procedure may cause additional vessel damage, resulting in extension of the hematoma and additional stenting in the distal part. Smith et al. (2) reported that during chronic total occlusion PCI, the subintimal transcatheter withdrawal (STRAW) technique, involving aspiration through a microcatheter to decompress the subintimal space, is effective to create the re-entry to the true lumen (Stingray System, Boston Scientific Corp., Natick, Massachusetts). We applied this aspiration technique to reduce subintimal hematoma volume after sealing the entry site by stenting without creating a re-entry.

**Learning Objectives**

• To show that coronary dissection caused by iatrogenic intimal damage or spontaneous intimal damage may lead to acute coronary occlusion.
• To determine the mechanism and treatment of this acute coronary occlusion caused by coronary dissection.
• To show that the STRAW technique is the most promising treatment for this type of acute coronary occlusion because it reduces the volume of hematoma in the subintimal space after sealing of the entry site by stenting without creating a re-entry.

## History of Presentation

**Figure 1:**
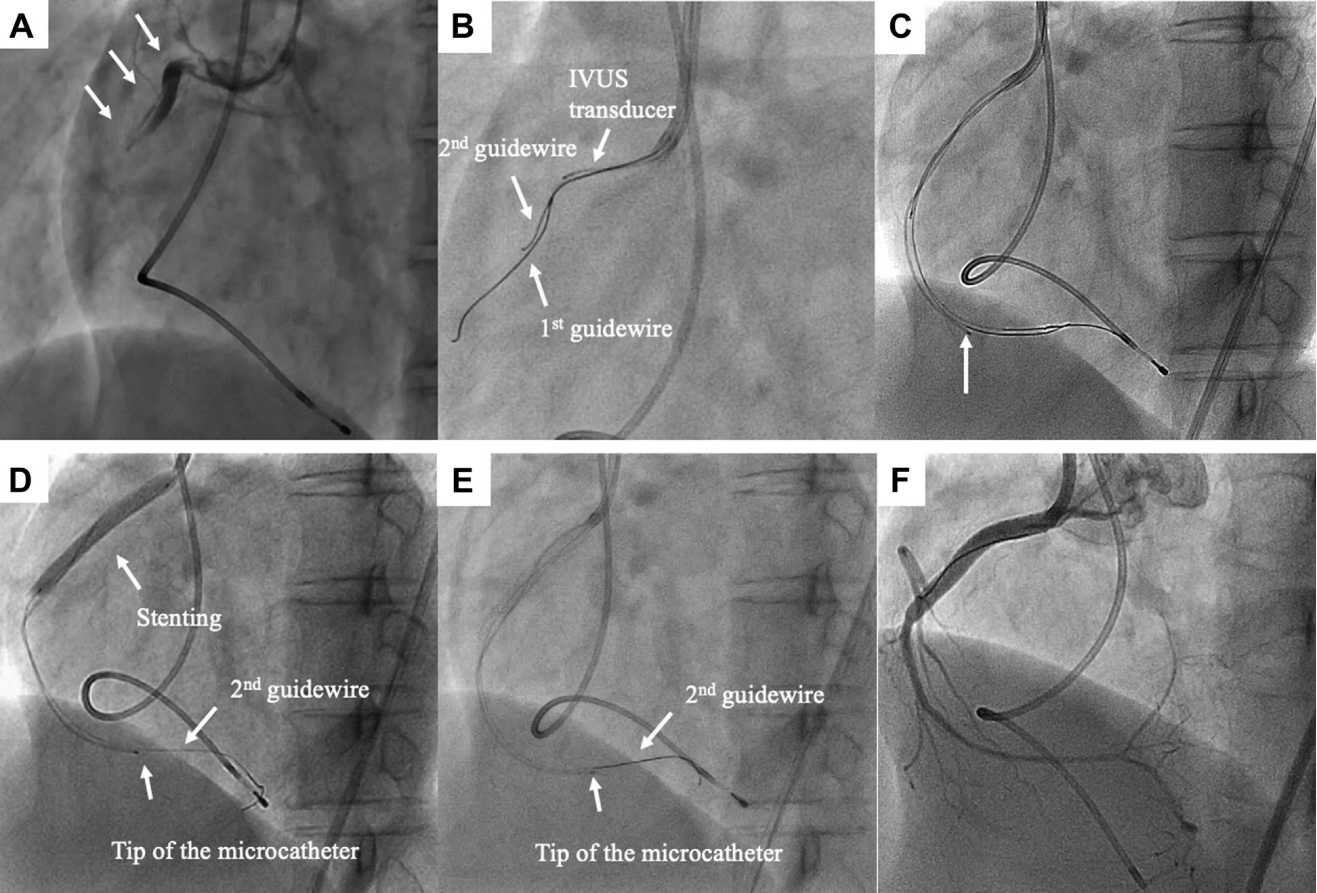
Angiographic Images **(A)** The ostium of the right coronary artery (RCA) was occluded by spiral dissection **(arrows)**. **(B)** The second guidewire was navigated into the true lumen under intravascular ultrasound (IVUS) observation. **(C)** The microcatheter **(arrow)** was inserted into and aspirated the blood from the subintimal space. **(D)** The stent was implanted to seal the entry site of the subintimal space. **(E)** Aspiration was continued after stent implantation. **(F)** TIMI flow grade 3 was finally obtained.

A 40-year-old woman was admitted to another hospital with sudden-onset chest pain at rest. The troponin I level was elevated to 1.53 ng/ml. Echocardiography revealed mild hypokinesis at the inferior wall of the left ventricle and ejection fraction 50%. She was diagnosed with acute coronary syndrome without persistent ST-segment elevation. After administration of loading doses of dual antiplatelet agents (200 mg aspirin, 20 mg prasugrel hydrochloride), she underwent emergency CAG via the right radial artery. Vasospastic angina was suspected as CAG showed a normal coronary artery. Injection of 20 mg acetylcholine into the right coronary artery (RCA) was performed to induce coronary spasm. Immediately after acetylcholine administration, the coronary artery was completely occluded from the ostium of the RCA, and angiography suggested spiral dissection (Figure 1A). Intracoronary injection of nitrate could not recanalize the occluded lesion. Shock developed, and an intra-aortic balloon pump was inserted for hemodynamic stabilization. Because the subintimal space may have been sufficiently enlarged to severely collapse the true lumen, it was difficult to insert the guidewire into the true lumen to recanalize the occluded lesion. She was transferred to our hospital to recanalize the occlusion.

## Differential Diagnosis

Occlusion of the coronary artery from the ostium of the RCA was suspected to have been caused by coronary spasm. Coronary angiography suggested spiral dissection from the ostium of the RCA.

## Management: PCI Procedure

**Figure 2:**
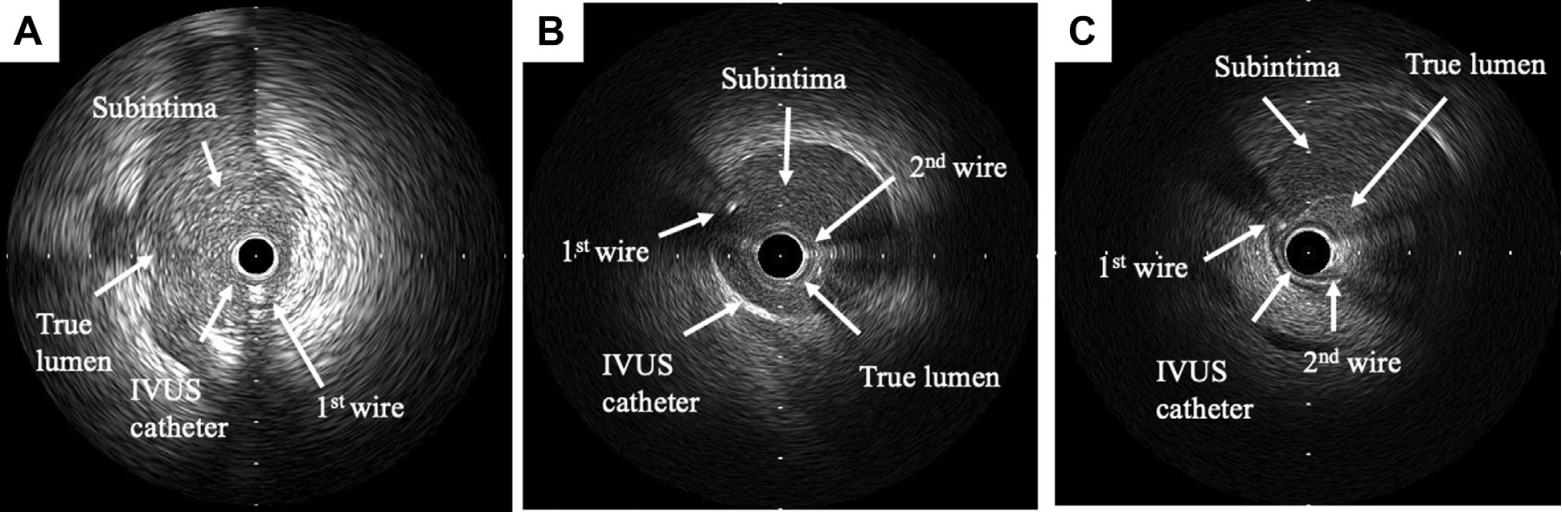
Cross-Sectional IVUS Images of Advancing the Guidewire Into the True Lumen **(A)** The first guidewire entered into the subintimal space and the subintimal huge hematoma collapsed the true lumen. **(B)** The second guidewire was navigated into the true lumen, which was considerably collapsed at the proximal site of the RCA. **(C)** At the distal part of RCA, the true lumen was completely collapsed. Abbreviations as in Figure 1.

**Figure 3:**
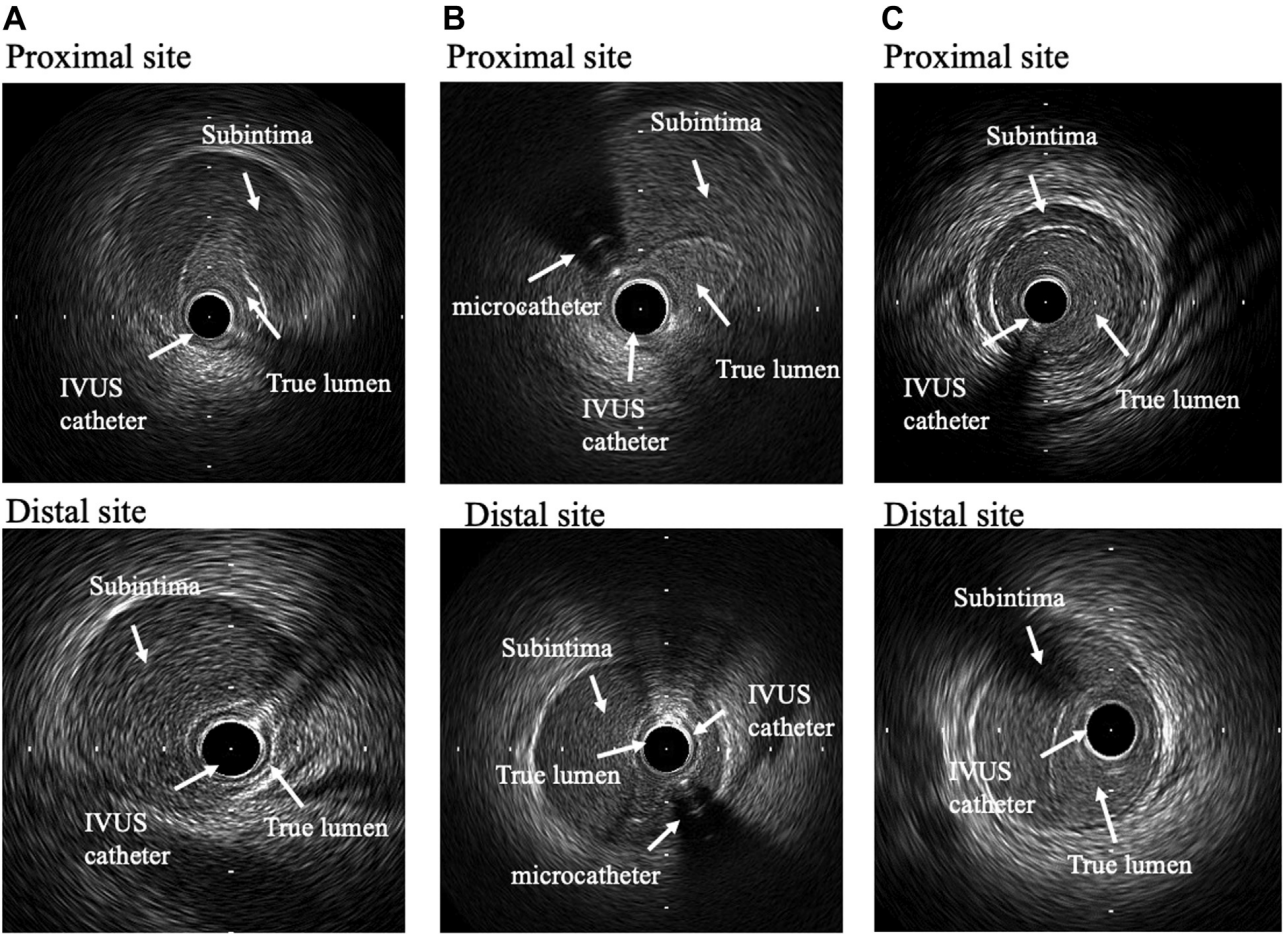
IVUS Images in the Series of Proximal and Distal RCA Lesions **(A)** Before aspiration. **(B)** After aspiration without sealing the entry site. **(C)** After aspiration with sealing the entry site by stenting. The true lumen area was significantly enlarged by aspiration after stenting at the proximal site (before aspiration, after aspiration, and after aspiration with stenting: 2.9, 2.6, and 5.2 mm^2^, respectively) and at the distal site (before aspiration, after aspiration, and after aspiration with stenting: 1.4, 1.9, and 3.9 mm^2^, respectively). Abbreviations as in Figure 1.

**Figure 4:**
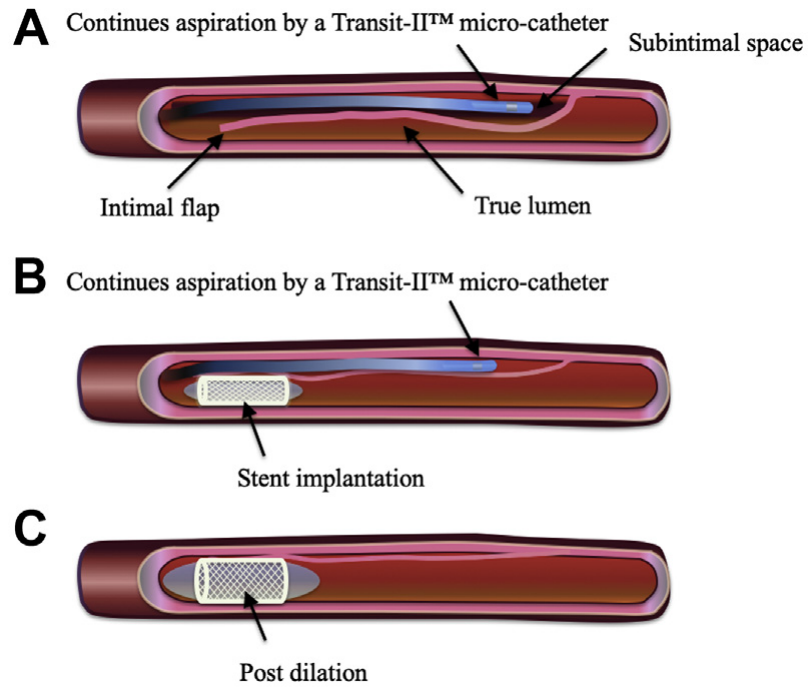
Aspiration Technique **(A)** A Transit-II 2.3-F microcatheter was inserted into the subintimal space and continuous aspiration was performed throughout the subsequent procedures. **(B)** The entry site was sealed by the stent to aspirate the residual hematoma. **(C)** The microcatheter was pulled out, followed by the 4.0-mm balloon after dilation to completely seal the entry site.

Emergency PCI was performed via the right femoral artery approach with the use of an 8-F guide catheter (Launcher JR 4.0, Medtronic AVE, Santa Rosa, California) for intravascular ultrasound (IVUS)–guided wiring. The first guidewire (SION wire, Asahi Intec Co., Aichi, Japan) was advanced into the occluded lesion without resistance. IVUS examination showed that the first guidewire entered the subintimal space and that the true lumen was collapsed by a huge subintimal hematoma resulting in RCA occlusion (Figure 2A). Under IVUS observation from the subintimal space, the second guidewire (Fielder FC wire, Asahi Intec Co.) was navigated into the true lumen (Figure 1B). IVUS examination showed that the second guidewire passed through the true lumen from proximal to distal, but the true lumen was completely collapsed by the huge subintimal hematoma at the distal part (Figures 2B and 2C). There was an entry site to the subintimal space proximal to the RCA, but there was no distal re-entry site. A 2.3-F microcatheter (Transit-II, Johnson and Johnson, Bridgewater, New Jersey) was inserted into the subintimal space, the tip of which was located at the distal site of the RCA. Under negative pressure from the PCI indeflator, the Transit-II microcatheter aspirated ∼10 cc of blood from the subintimal space (Figure 1C), but the true lumen was only slightly enlarged owing to the entry site patency. To aspirate the residual hematoma, the entry site was sealed by stent implantation (3.5 × 38 mm Resolute integrity stent; Medtronic AVE) (Figure 1D). While slowly inflating the stent balloon, the microcatheter was pulled back 1 to 2 cm proximally to check the resistance and avoid becoming trapped between the vessel wall and stent struts. After stent implantation at 8 atm (Figure 1E), the true lumen was confirmed by IVUS examination to be enlarged by continuous aspiration of blood from the subintimal space (Figure 3). CAG showed TIMI flow grade 1 immediately after stenting but TIMI flow grade 3 after continuous aspiration from the subintimal space (Figure 1F). After confirming TIMI flow grade 3, the microcatheter was pulled out of the coronary artery, followed by the 4.0-mm noncompliant balloon after dilation to 20 atm to completely seal the entry site (Figure 4). The intraaortic balloon pump was pulled out the day after PCI because the hemodynamics had recovered. The maximum creatinine kinase level was 2,280 IU/l.

## Follow-Up

The patient was discharged on postoperative day 13 and was asymptomatic at follow-up 7 months later.

## Discussion

Creation of a distal re-entry site is widely performed to solve coronary flow disturbance due to compression of the true lumen by subintimal hematoma. However, this method does not always work and is accompanied by the risk of further vessel damage. The present aspiration technique after sealing of the entry site by stenting is a more promising method because the hematoma volume can be reduced by directly collecting blood without additional vessel damage. However, when performing this technique, it is necessary to exclude the possibility of complete coagulation of hematoma blood by IVUS examination, and to avoid the microcatheter becoming stuck between the vessel wall and stent struts by pulling back the microcatheter slightly proximally to check the degree of resistance while slowly inflating the stent balloon. This procedure is acceptable because stent-traps for microcatheters are commonly used during coiling in neurointerventional radiology (3). If the aspiration method does not work after sealing the entry site by stenting, cutting balloon dilation at a distal site should be considered to create a re-entry at this point. We considered these points in the present case and obtained promising TIMI flow grade 3 with the use of this aspiration method without additional mechanical damage to the distal site, such as the creation of a re-entry or other additional stenting at the distal part.

## Conclusions

We report a case of acute coronary occlusion by huge hematoma formation in the subintimal space that was successfully recanalized with aspiration of the hematoma after sealing the entry site by stenting.

## Funding Support And Author Disclosures

All authors have reported that they have no relationships relevant to the contents of this paper to disclose.
